# Examining the variability of multiple daily symptoms over time among individuals with multiple long-term conditions (MLTC-M/multimorbidity): An exploratory analysis of a longitudinal smartwatch feasibility study

**DOI:** 10.1177/26335565221150129

**Published:** 2023-01-18

**Authors:** Khalid Kazi, Syed Mustafa Ali, David A Selby, John McBeth, Sabine van der Veer, William G Dixon

**Affiliations:** 1 Northern Care Alliance NHS Foundation Trust, Salford, UK; 2 Centre for Epidemiology Versus Arthritis, Division of Musculoskeletal and Dermatological Sciences, Manchester Academic Health Science Centre, 5292 The University of Manchester, Manchester, UK; 3 Centre for Health Informatics, Division of Informatics, Imaging and Data Science, Manchester Academic Health Science Centre, 5292 The University of Manchester, Manchester, UK; 4 NIHR Manchester Biomedical Research Centre, Manchester NHS Foundation Trust, Manchester, UK

**Keywords:** Multiple long-term conditions (multimorbidity), smartwatch, patient-generated health data, multiple daily symptoms, symptom variability, fatigue

## Abstract

**Introduction:**

People living with multiple long-term conditions (MLTC-M) (multimorbidity) experience a range of inter-related symptoms. These symptoms can be tracked longitudinally using consumer technology, such as smartphones and wearable devices, and then summarised to provide useful clinical insight.

**Aim:**

We aimed to perform an exploratory analysis to summarise the extent and trajectory of multiple symptom ratings tracked via a smartwatch, and to investigate the relationship between these symptom ratings and demographic factors in people living with MLTC-M in a feasibility study.

**Methods:**

‘Watch Your Steps’ was a prospective observational feasibility study, administering multiple questions per day over a 90 day period. Adults with more than one clinician-diagnosed long-term condition rated seven core symptoms each day, plus up to eight additional symptoms personalised to their LTCs per day. Symptom ratings were summarised over the study period at the individual and group level. Symptom ratings were also plotted to describe day-to-day symptom trajectories for individuals.

**Results:**

Fifty two participants submitted symptom ratings. Half were male and the majority had LTCs affecting three or more disease areas (N = 33, 64%). The symptom rated as most problematic was fatigue. Patients with increased comorbidity or female sex seemed to be associated with worse experiences of fatigue. Fatigue ratings were strongly correlated with pain and level of dysfunction.

**Conclusion:**

In this study we have shown that it is possible to collect and descriptively analyse self reported symptom data in people living with MLTC-M, collected multiple times per day on a smartwatch, to gain insights that might support future clinical care and research.

## Introduction

The prevalence of multiple long-term conditions (multimorbidity) (MLTC-M), defined as having two or more long term conditions concurrently,^1^ is increasing globally.^2^ MLTC-M is associated with significant economic burden with £7 out of every £10 already spent on MLTC-M in 2012 in England.^3^ MLTC-M has in recent years been recognized as a priority area for research.

Studying MLTC-M is challenging because of the high number of different combinations of diseases, with associated symptoms that change in severity, importance and impact through time. Increased symptom burden has been associated with poorer quality of life.^4–6^ Symptoms shape the personal experience of living with disease, help clinicians to diagnose, treat and monitor disease through time, and guide self-management in between consultations. They also inform research in the understanding of disease, the factors that influence the onset or exacerbation of symptoms, and assessment of optimal management. Symptom experience, however, is complex and idiosyncratic with symptoms influencing each other in ways that are not clearly characterized.^7–9^

Prior cross-sectional studies of symptom burden have assessed single symptoms in a general MLTC-M population,^10–12^ have looked at multiple symptoms within a specific demographic^5,6,13^ or have examined multiple symptoms in a general MLTC-M population.^14,15^ While some studies look at MLTC-M longitudinally, for example examining how symptom burden correlates with subsequent mortality^16^ or how symptoms change at discrete intervals of, say, 6 months,^17^ few studies have been able to look at the day-to-day patterns of symptoms – which is of course how people experience living with disease.

Mobile health (mHealth) approaches offer a potential solution. If symptoms are collected regularly, the resultant time series data can be used to dynamically study short and long term trends in multiple symptoms simultaneously.^18^ Additionally, time series data can also be used to both interrogate causality and, theoretically, forecast future disease activity. mHealth also has the advantage of scalability, with the potential to collect data from large numbers of participants given the high population uptake of such consumer devices, including in older populations.^19,20^ Smartphones and smartwatches have been successfully used to track specific pre-existing health conditions, such as chronic pain,^21^ rheumatoid arthritis^22^ and heart failure^23^ and also to detect the onset of health conditions such as Covid 19.^20^ Wearable devices such as smartwatches have the additional benefit of combining survey questions with passively collected sensor data which can be leveraged to detect changes in activity levels and physiological measures such as heart rate and rhythm.^24,25^

We have conducted a smartwatch feasibility study (Watch Your Steps) in order to explore how we might harness the potential of smartwatches to explore longitudinal symptom patterns in MLTC-M populations. Participants with MLTC-M submitted their daily ratings of a range of symptoms via a consumer smartwatch touch face for 90 days. In our previous publication we reported on the feasibility of using smartwatches to track multiple symptoms per day, demonstrating good engagement with around 45% of all potential data points (up to approximately 1800 per participant over the 90 days) reported during the three month study period.^26^ In the present post hoc analysis, we aim to perform an exploratory analysis of the daily data collected in Watch Your Steps to examine the extent, variability and patterns of longitudinal symptoms in people living with MLTC-M. The specific objectives are to:

1. Summarise the extent of common symptoms over the study period.
2. Investigate the relationship between symptoms and key demographic characteristics including number of disease areas.
3. Illustrate day to day changes in and relationships between symptoms by using illustrative examples of selected participants.

## Methods

### Study design

“Watch Your Steps” is a smartwatch-based longitudinal feasibility study, collecting multiple daily survey questions and weekly active tasks over 90 days from people living with MLTC-M. The study design and participant recruitment has been described in full in our previous publication,^26^ and is summarised below.

### Participant eligibility and recruitment

Adults (aged 18 and above) with more than one clinician-diagnosed long-term condition were eligible to take part in the study. Participants were recruited from five specialist outpatient clinics at a local teaching hospital; one community GP surgery; and two local patient and public involvement and engagement groups, all in Greater Manchester. Interested participants were screened by telephone for eligibility, then eligible participants were invited to an on-boarding event where they were consented, instructed on how to use their smartwatch and provided with a copy of the app user guide. We aimed to recruit 60 participants to examine the study’s primary aim of acceptability and feasibility, although the onset of the pandemic meant recruitment was curtailed to 52 participants.

### Data collection

**Figure 1.**
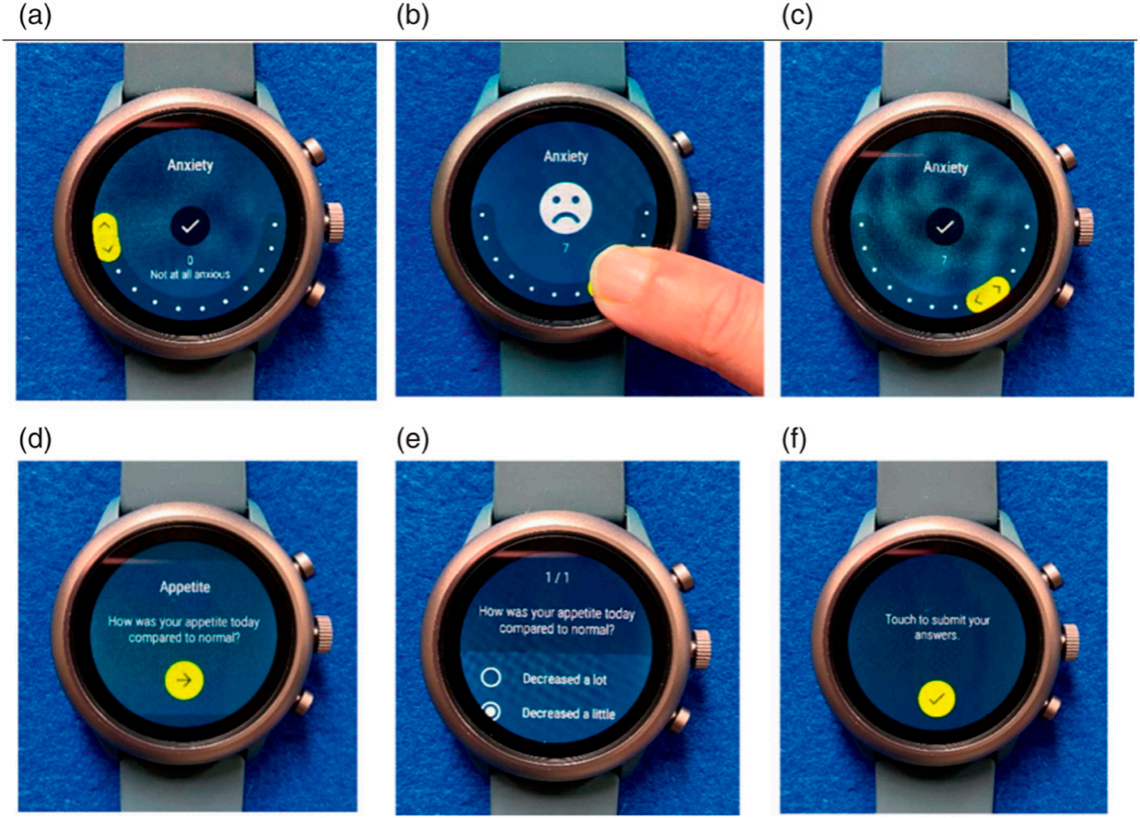
Images of the smartwatch face showing different input methods and their steps. (a) Radial interface for anxiety (a symptom question with a numerical rating scale response). (b) Moving selector on the radial interface showing a dynamic emoticon. (c) Submitting response by tapping the tick mark. (d) Wording of the appetite question (a symptom with a categorical response). (e) Selection of a categorical response option. (f) Submitting response by tapping the tick mark.

Participants were provided with loaned Fossil Sport smartwatches which were pre-loaded with the study app (See Figure 1). Participants completed two baseline questionnaires, one on the web and the other on the study smartwatch, including questions about the disease areas affected by their long-term conditions (for example, a patient with asthma and eczema would select ‘heart and lung’ and ‘skin’ disease areas).

Subsequently, using the smartwatch, participants were prompted to complete daily and weekly survey questions and active tasks as listed in Supplementary Table 1. Each study participant received a prompt at the specified day/time to complete either core symptom questions (for all participants), or disease area-specific symptom questions. The seven core symptoms were pain, fatigue, wellbeing, mood, stress, function, and sleep quality; the disease area-specific questions included questions such as breathlessness for ‘heart and lung’, itchiness for ‘skin’ and ‘kidney’, drowsiness for ‘kidney’ and anxiety for ‘mental health’. Participants were typically asked to report around 20 responses per day over the 90 days, equating to around 1800 responses in total. Responses to questions were collected either on a numerical rating scale or as categorical responses as demonstrated in Figure 1. The smartwatch also collected continuous passive data on physical activity and heart rate from its gyroscope, accelerometer and photoplethysmography sensors. Analysis of these sensor data will be reported in the future.

The study was approved by the National Health Services Research Ethnics Committee and the Health Research Authority’s approval (19/WM/0307).

### Data analysis

To estimate the overall extent of different symptoms (Objective 1), we first calculated the daily symptom rating per participant as the sum of symptom ratings for a particular day divided by the total number of ratings given that day (for example, when pain was reported multiple times per day). We then used these daily ratings to calculate average symptom ratings per participant for the overall study period as the sum of daily participant symptom ratings divided by the total number of study days on which a participant submitted data to account for missing data. Finally, we used these ratings to calculate overall mean symptom ratings across all participants and for subgroups based on the affected disease area.

To explore the relationship between symptoms and demographic factors (Objective 2), we focused on fatigue as it was the symptom that participants considered the most useful to track^26^ and had the highest level of completeness (See Supplementary Figure 1). We plotted the distribution of participant-level average symptom ratings for fatigue across the study period as a dotplot and then coloured the plots according to gender, age and number of disease areas affected. Due to the nature of our study, the dataset is underpowered to conduct robust null hypothesis statistical testing to detect differences between groups. However, exploratory student’s t-test were performed and results should be interpreted with caution. To investigate the relationship between fatigue and other symptoms, we plotted the patient-level daily symptom ratings for fatigue against the daily ratings for the 10 other symptoms. There are therefore multiple observations per participant. We elected to analyse this at the scale of daily ratings rather than over the study period as this ought to give more insight into how symptoms are linked dynamically. We then calculated Pearson product moment correlation coefficients between the patient level daily ratings for fatigue and the other 10 symptoms again including multiple observations per participant.

To illustrate day-to-day changes in daily symptom ratings (Objective 3), we focused on three symptoms; pain, mood and fatigue. Our rationale for selecting these symptoms was that a) they were previously reported as prevalent amongst people with MLTC-M,^15^ b) they have higher completeness than other symptoms tracked during the study,^26^ and c) there are clinically plausible associations between the symptoms. In illustrative examples, data points were connected by a straight line. For days where data was missing, the line connects the points for which data was available. A seven-day moving average was calculated and plotted to help elucidate longer term trends in symptoms. The intention of this objective was to show the potential of measuring daily longitudinal data in real-time, allowing a description of how symptoms align, and potentially interact, with one another temporally.

All data were analysed and visualized using R (R Core Team., 2021).

## Results: Characteristics of the study population

A total of 52 participants entered data on a median of 62 out of the 90 days of the study period and the overall completion rate of symptom questions was 45% (interquartile range (IQR) 23–67%) (Supplementary Figure 2). The majority of participants (62%) were aged over 50 and had confirmed LTCs affecting three or more disease areas (64%), with the musculoskeletal system being most commonly affected (67%) (Table 1). Males and females were equally represented.

**Table 1.** Demographic characteristics of the study participants.

		n	%
Age	18-29	9	17
	30-39	3	6
	40-49	8	15
	50-59	15	29
	60-69	13	25
	70-79	4	8
Sex	Female	26	50
	Male	26	50
Disease areas	Mental health	20	39
	Bone, joint or muscle	35	67
	Skin	22	42
	Heart or lung	22	42
	Stomach or bowel	20	39
	Kidney	7	14
	Endocrine	18	35
	Neurological	8	15
	Other	16	31
Total number of disease areas affected*	1 or 2	19	37
	≥3	33	64

* All participants were confirmed as having two or more long-term conditions during eligibility screening. The number of disease areas affected refers only to the specific named disease areas listed here.

### The extent of different symptoms over the study period

Table 2 shows the overall mean symptom ratings across participants, stratified by disease areas affected. For all symptoms except mood, wellbeing and sleep, a higher rating related to a worse experience of that symptom. The core symptom rated as worst overall was fatigue, followed by pain. Participants with mental health conditions numerically had the lowest mood but also had the highest mean pain and fatigue scores overall.

**Table 2.** Overall mean symptom ratings across participants, stratified by affected disease area. The lower portion of the table displays the results for examples of disease area specific questions.

	All	Bone & Joint	Heart & Lung	Skin	Stomach & Bowel	Mental	Endocrine	Neurological	Kidney
Participants N	52	35	22	22	20	20	18	8	7
Core symptoms (mean score over the study period)									
Fatigue	**4.2**	4.8	4.3	4.5	4.7	5.3	3.2	4.8	3.0
Pain level	3.5	4.3	3.7	3.4	3.7	4.6	3.1	4.6	2.9
Function	3.2	3.9	3.2	3.6	3.6	4.3	2.8	4.8	2.6
Stress	3.0	3.3	2.5	3.0	2.9	4.2	2.7	2.9	2.0
Mood	6.6	6.3	7.2	6.6	6.6	**5.5**	7.0	6.5	7.1
Wellbeing	6.5	6.2	6.8	6.2	6.0	5.3	6.9	5.5	6.7
Sleep quality	5.8	5.6	5.8	5.7	6.0	5.2	5.9	5.3	5.6
Disease area specific symptoms (mean score over the study period)									
Breathlessness			2.5						
Itch				2.3					1.4
Anxiety						4.3			
Drowsiness									2.4


In this analysis we report on all core symptoms: mood, sleep quality, pain level, fatigue, stress, function and wellbeing, and four examples of “disease area-specific” symptoms: average breathlessness, average itch, anxiety and drowsiness. Worst itch and worst breathlessness are not reported as they were highly correlated to the average itch and average breathlessness ratings, respectively, while other symptoms such as appetite, bowel habit and morning stiffness were reported on different scales.

Our exploratory analysis indicated that there was little indication of a readily apparent difference, between symptom ratings based upon age or sex (Supp Figure 3A and C). These figures increased comorbidity (3+ disease areas affected) may be associated with poorer ratings for all symptoms and the most marked discrepancy appeared to be for functional impairment (see supplementary Figure 3B).

### Relationship between self-reported fatigue and key demographic characteristics

**Figure 2.**
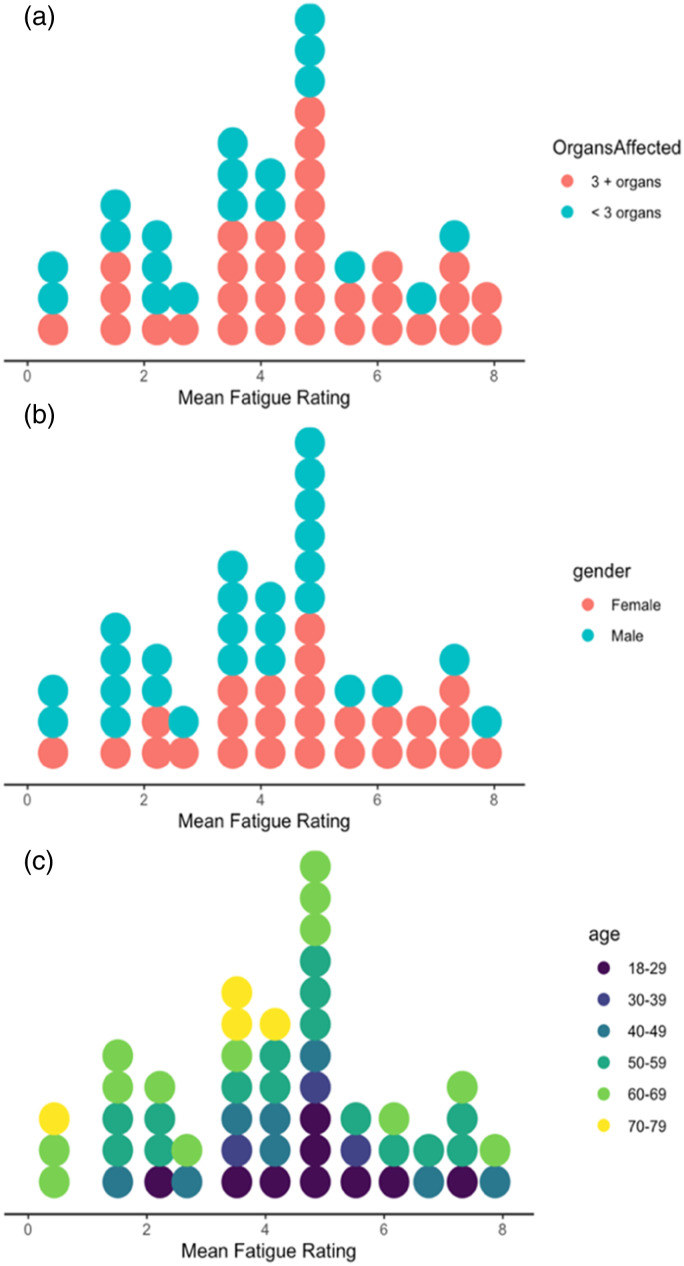
Dot plots illustrating the distribution of mean fatigue ratings over the study period coloured by (a) the number of disease areas affected, (b) gender and (c) age.

Mean fatigue ratings over the study period for individual participants appeared to be approximately normally distributed with a wide range observed (Figure 2).

Patients with more disease areas affected by long term conditions (three or more disease areas, mean = 4.6/10, less than three disease areas, mean = 3.5/10, P = 0.052) (panel A) and females (female = 4.7/10, male = 3.7/10, P = 0.078) (panel B) tended to have higher fatigue ratings but only to a limited extent. No clear relationship between age and fatigue ratings can be observed (panel C). Visually, individuals with conditions affecting mental health or the musculoskeletal system show a signal to higher fatigue levels (See Supplementary Figure 4). Please note, our study was not powered to detect statistical differences and the above statistical tests are exploratory.

Across participants, the strongest positive correlations were seen between the daily fatigue rating and daily ratings for function (R = 0.68) and pain (0.65). Modest negative correlations were seen between the daily fatigue rating and daily ratings for wellbeing (R = -0.49), sleep quality (-0.47) and mood (-0.43) (See Supplementary Figure 5).

### Day to day changes in symptom ratings

**Figure 3.**
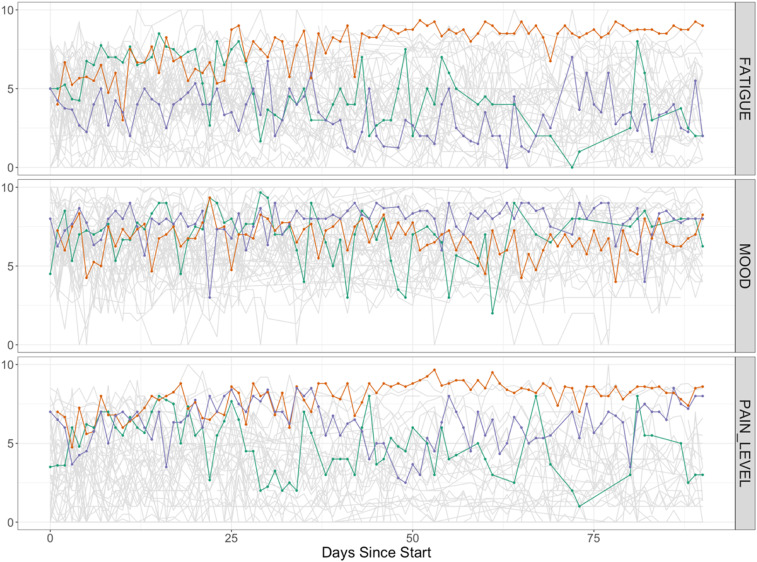
Line plots illustrating summarises the trajectories of symptom ratings for fatigue (top panel), mood (middle panel) and pain (bottom panel) over the study period. Each line represents an individual within the cohort with the three coloured lines highlighting three individuals chosen at random for clarity. For pain and fatigue, a high score indicates a negative experience of that symptom and for mood the opposite is true.

We observed a wide variation in the trajectories of symptom ratings over time (Figure 3). We can see that whilst the extremes of the scale were used for all symptoms, mood was less commonly rated as worse (i.e., less) than 5 out of 10.

### Relationships between different daily symptoms

**Figure 4.**
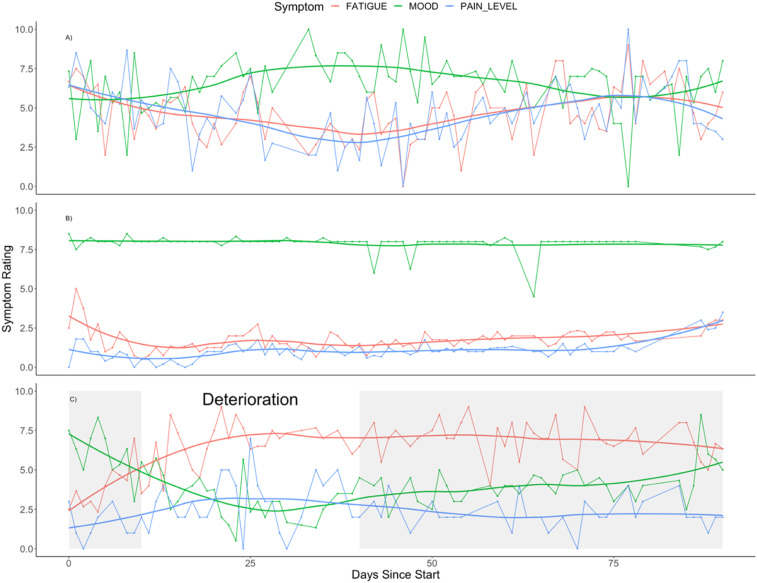
Line plots summarizing the trajectory of fatigue (red), mood (green) and pain (blue) over the study period for three participants. Each panel represents a different study participant (A, B and C). The dotted lines represent the raw daily symptom ratings and the smoothed lines represent the 7 day moving average. For pain and fatigue, a high score indicates a negative experience of that symptom and for mood the opposite is true.

An important potential benefit of symptom tracking arises from the ability to see day-to-day changes within individuals, as well as the (potentially causal) relationships between different symptoms. Figure 4 depicts how ratings for mood, pain and fatigue for three individuals varied over the study period.

Firstly, we can appreciate general trends and differences between individuals. From the moving average (smooth lines) for fatigue (red) and mood (green) we can see that participant C has generally much higher levels of fatigue than participant B and much lower mood levels than either participant A or B. Ratings for fatigue remain roughly stable throughout the study period for participant B, whilst for participants A and C there is a clear change in the levels of fatigue.

Secondly, we can appreciate differences in the day-to-day volatility of symptom ratings. Looking more closely at the daily symptom ratings (dotted lines) we can see that whilst participant B enjoys relative stability in terms of their symptom ratings, participants A and C experience a great deal more volatility (noisiness of the dotted line around the smooth line), this is most clearly apparent examining the green line for mood for participant A and contrasting that with participant B.

Finally, we can gain insight into the individual participant’s experience of symptoms over the study period in terms of general patterns, moments of interest and apparent correlations between symptoms. Between days 15 and 65 of the study, participant A’s pain and fatigue are relatively improved and during this interval their mood is good. However, at around day 75 there is a spike in both pain and fatigue which is associated with a worsening in mood. For participant B, a moment of interest is captured around day 65 where they experience a marked reduction in mood which appears to be unrelated to either pain or fatigue levels. Days 1 to 30 for participant C capture a period of deterioration, which includes an increase in fatigue ratings accompanied by a marked deterioration in mood and a modest increase in pain. It takes some weeks before this participant’s mood rating improves, and their fatigue rating remains high. From the graph there is an impression of a negative correlation between this participant’s fatigue ratings and their mood although it is not clear whether a deterioration in one precedes, or indeed causes, deterioration in the other.

## Discussion

Consumer technology has been championed as an opportunity to track, monitor and investigate longitudinal symptoms in people living with MLTC-M,^27^ and is now seen as a priority area.^18^ In our previous publication about engagement in Watch Your Steps,^26^ one third of participants provided data on a nearly daily basis, and the overall completion rate of symptom questions was 45% (interquartile range (IQR) 23–67%).

In the present analysis, we have demonstrated the ability to study a range of daily self-reported symptoms in people living with MLTC-M over three months and to observe how these symptoms change over time. In a cohort of people living with different combinations of LTCs, we were able to do this by tracking a range of core symptoms collected across all participants, plus some additional specific symptoms by disease area.

Among the range of self-reported symptoms, pain and fatigue were two commonly reported symptoms and considered useful to track daily by people with MLTC-M.^26^ Our exploratory analysis suggested fatigue to be the symptom with the highest average score over three months, followed by pain. Also, fatigue was strongly positively correlated with pain, level of perceived dysfunction and was negatively correlated with mood and sleep quality. Our findings add to and complement existing knowledge about fatigue in MLTC-M. Though prevalence of MLTC-M is higher among older adults,^28^ there was no clear tendency for older people to report worse symptoms than younger adults. However, higher burden of fatigue and pain has previously been observed across all disease areas among older adults.^29,30^ Fatigue has been reported as a major symptom in conditions with chronic pain (such as in fibromyalgia and chronic fatigue syndrome), and having greater impact on people’s functioning.^31^ Other studies, including large-scale population surveys, have found that people with more morbidities have reported more symptoms, subsequently reporting more impairment,^15^ and that severe fatigue increases with increasing numbers of chronic diseases^32,33^ – whilst our study was unpowered and exploratory, these findings are in line with our own preliminary results. In addition, fatigue is important in managing chronic conditions^34^ as it lowers patients’ motivation to actively engage in rehabilitation programs.^35^

For managing multiple symptoms, a constant challenge for researchers and clinicians has been understanding how symptoms change through time; and how individuals respond to interventions.^36–38^ Consumer devices introduce many opportunities for health research including recruiting at scale and the ability to contribute richer and more frequent data regularly from the home.^39^ Moreover, traditional self-reported questionnaires that consider how the participant has been in the last X days, weeks or months, is prone to recall error. Prospectively collected daily data avoids such limitations. Our analysis provides an early, yet important first step in demonstrating such opportunities from consumer devices for MLTC-M research. We have provided a first view into how multiple symptoms change day-by-day, exploring gradual trends over periods of weeks, more acute changes between days, and suggestions of correlations between different symptoms. It is plausible to imagine that tracking temporal changes in symptoms in MLTC-M might detect early – and ultimately prevent – deterioration with timely interventions. In the future, there is the opportunity for the *prediction* of events, in turn leading to possible just-in-time adaptive interventions,^40^ delivered either by new care pathways or as digital interventions via the same device. Day to day fluctuations in affect have previously been studied in patients with depression and have shown some promise in predicting episodes of clinical depression.^41^

Our exploration of time-varying symptoms was mostly descriptive, with visual case studies allowing the reader to observe and interpret graphs of changing symptoms through time. We were not trying to present representative or common patterns, but instead show through selected illustrative examples that real-time tracked data provide insight around potentially correlated symptoms. The human eye can appreciate these associations and patterns in visual graphs, yet it is hard to summarise across a population. There is an important research agenda emerging about how best to describe and summarise changing patterns through time, hence demanding advanced analytical methods,^42^ and acknowledging that current exploratory models (e.g., linear, cause-and-effect approach to outcomes) are not sufficient to study multimorbidity.^43^ Modelling the time-series data will be important as we seek to answer clinically relevant questions about causal relationships, for example how interventions or wider contextual or environmental factors such as physical activity could influence changing symptoms. Indeed, we intend to conduct further analyses of our preliminary Watch Your Steps data to examine how patterns of physical activity assessed using raw sensor data (such as accelerometer, gyroscope and heart rate) relate to fatigue.

Despite the feasibility and benefits of tracking temporal changes in symptoms, there are some important limitations and questions that remain unanswered. The Watch Your Steps study was designed to evaluate the feasibility and acceptability of using smartwatches to study multimorbidity, and the ability to collect multiple symptoms per day across disease areas.^26^ Accordingly, it had a relatively small number of participants, who were sufficiently motivated and digitally literate, hence generalizability of symptom variability among MLTC-M population is limited. Furthermore, due to the nature of our dataset, we were underpowered to conduct robust and reliable statistical testing during our analysis. We do not know whether missing information was missing at random, or whether participants did not report because they were feeling particularly well or, conversely, particularly unwell. We limited our analyses to daily summaries of symptoms. It is important to note that within-day variability can also contribute to the burden of living with MLTCs. While we collected several symptoms multiple times per day, we did not extend the current analysis to look at this. Stratification by disease area was underpowered for robust conclusions yet provides an interesting ‘first look’ into the data. We know from other studies that a clear purpose of daily data collection, such as informing clinical consultations, has the potential to boost engagement through time because of a more direct benefit to participants.^22^ Our analysis of how different symptoms relate to fatigue at the population level was a compromise, conducting the analysis at the daily level captures how symptoms interact dynamically, but leads to multiple observations per participant and is therefore weighted more towards those participants who engaged with the study more. Disease areas were self-reported, which others have argued may be inaccurate. In the future, linking self-reported patient symptoms with clinician-reported data from electronic health records will not only verify diagnosis but will also support clinical decision making for better treatment and disease management. We are pioneering the integration of self-reported data into the NHS for people living with rheumatoid arthritis,^22^ and hope to expand out to other disease areas including MLTCs in time allowing more person-centered care. Lastly, we provided smartwatches for our participants: this is likely to be required in future studies in the near-term given the relatively low penetration of smartwatches in the MLTC population, which might then affect scalability.

## Conclusion

In conclusion, we have shown that it is feasible to summarise symptom burden by capturing day-to-day variations in symptoms using data collected via a smartwatch in individuals living with MLTC-M. Fatigue contributed the most to overall symptom burden. We observed a signal that people living with more conditions had more severe symptoms with poorer function. Importantly, we were able to see clearly how symptoms change day-to-day, something that has been elusive in the past. This new opportunity of tracking symptoms of multimorbidity alongside other data has the potential to transform self-management, clinical care and research, and could provide useful insight about the day-to-day fluctuations in diseases and their complex interactions.

## Supplemental Material

Supplemental Material - Examining the variability of multiple daily symptoms over time among individuals with multiple long-term conditions (MLTC-M/multimorbidity): an exploratory analysis of a longitudinal smartwatch feasibility study Click here for additional data file. Supplemental Material for Examining the variability of multiple daily symptoms over time among individuals with multiple long-term conditions (MLTC-M/multimorbidity): an exploratory analysis of a longitudinal smartwatch feasibility study by Khalid Kazi, Syed Mustafa Ali, David A Selby, John McBeth, Sabine van der Veer and William G Dixon in Journal of Multimorbidity and Comorbidity

Supplemental Material - Examining the variability of multiple daily symptoms over time among individuals with multiple long-term conditions (MLTC-M/multimorbidity): an exploratory analysis of a longitudinal smartwatch feasibility study Click here for additional data file. Supplemental Material for Examining the variability of multiple daily symptoms over time among individuals with multiple long-term conditions (MLTC-M/multimorbidity): an exploratory analysis of a longitudinal smartwatch feasibility study by Khalid Kazi, Syed Mustafa Ali, David A Selby, John McBeth, Sabine van der Veer and William G Dixon in Journal of Multimorbidity and Comorbidity

Supplemental Material - Examining the variability of multiple daily symptoms over time among individuals with multiple long-term conditions (MLTC-M/multimorbidity): An exploratory analysis of a longitudinal smartwatch feasibility study Click here for additional data file. Supplemental Material for Examining the variability of multiple daily symptoms over time among individuals with multiple long-term conditions (MLTC-M/multimorbidity): An exploratory analysis of a longitudinal smartwatch feasibility study by Khalid Kazi, Syed Mustafa Ali, David A Selby, John McBeth, Sabine van der Veer and William G Dixon in Journal of Multimorbidity and Comorbidity
