# Supplementary material for: Examining the variability of multiple daily symptoms over time among individuals with multiple long-term conditions (MLTC-M/multimorbidity): An exploratory analysis of a longitudinal smartwatch feasibility study
Source: J Multimorb Comorb. 2023 Jan 18;13:26335565221150129. doi: 10.1177/26335565221150129 (PMC9869202; doi:10.1177/26335565221150129)
Supplement: Supplemental Material - Examining the variability of multiple daily symptoms over time among individuals with multiple long-term conditions (MLTC-M/multimorbidity): an exploratory analysis of a longitudinal smartwatch feasibility study [file sj-pdf-1-cob-10.1177_26335565221150129.pdf]

Supplementary Figures

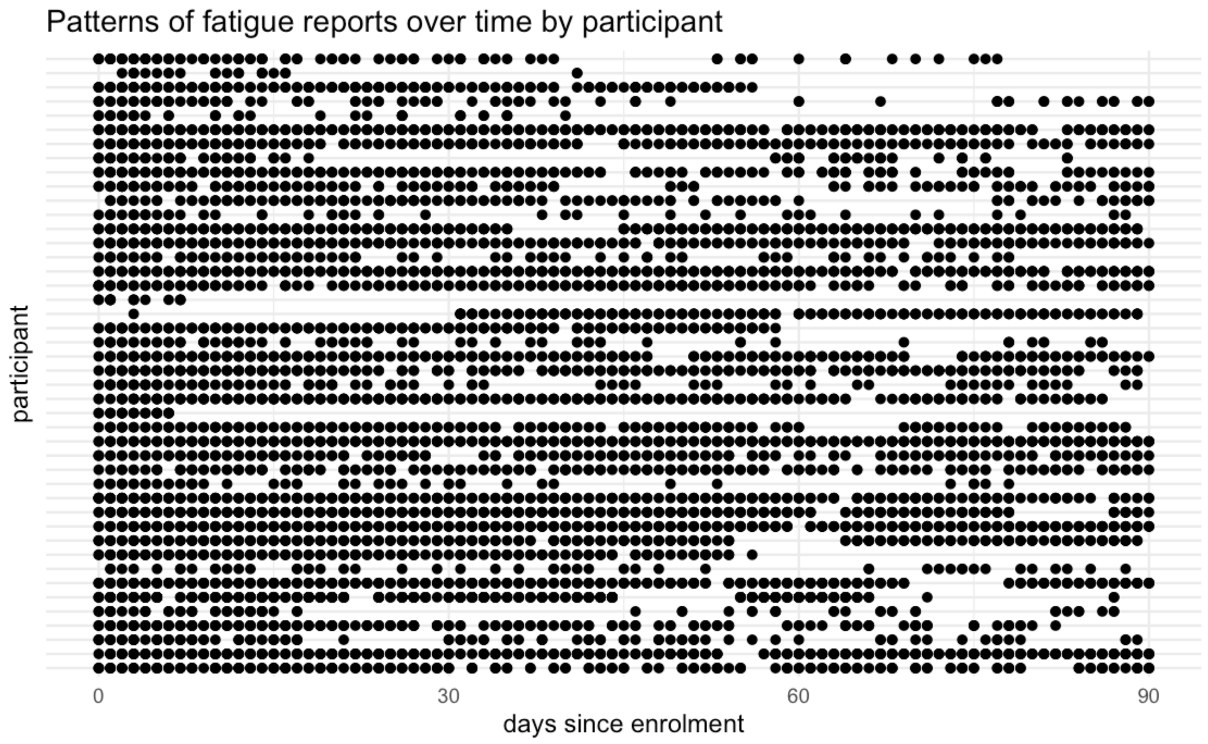

Supplementary Figure 1. Dot plot illustrating the patterns of engagement of all study participants with the daily ratings for fatigue. Each row represents an individual participant. A dot is present if the participant inputted at least one rating for fatigue on that day and absent if no ratings were inputted.

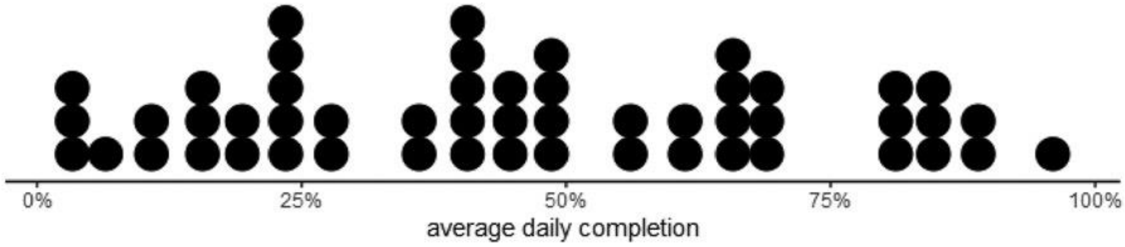

Supplementary Figure 2. Distribution of average daily completion rate of scheduled questions among the participants over the whole 90-day study period. Each dot represents one participant.

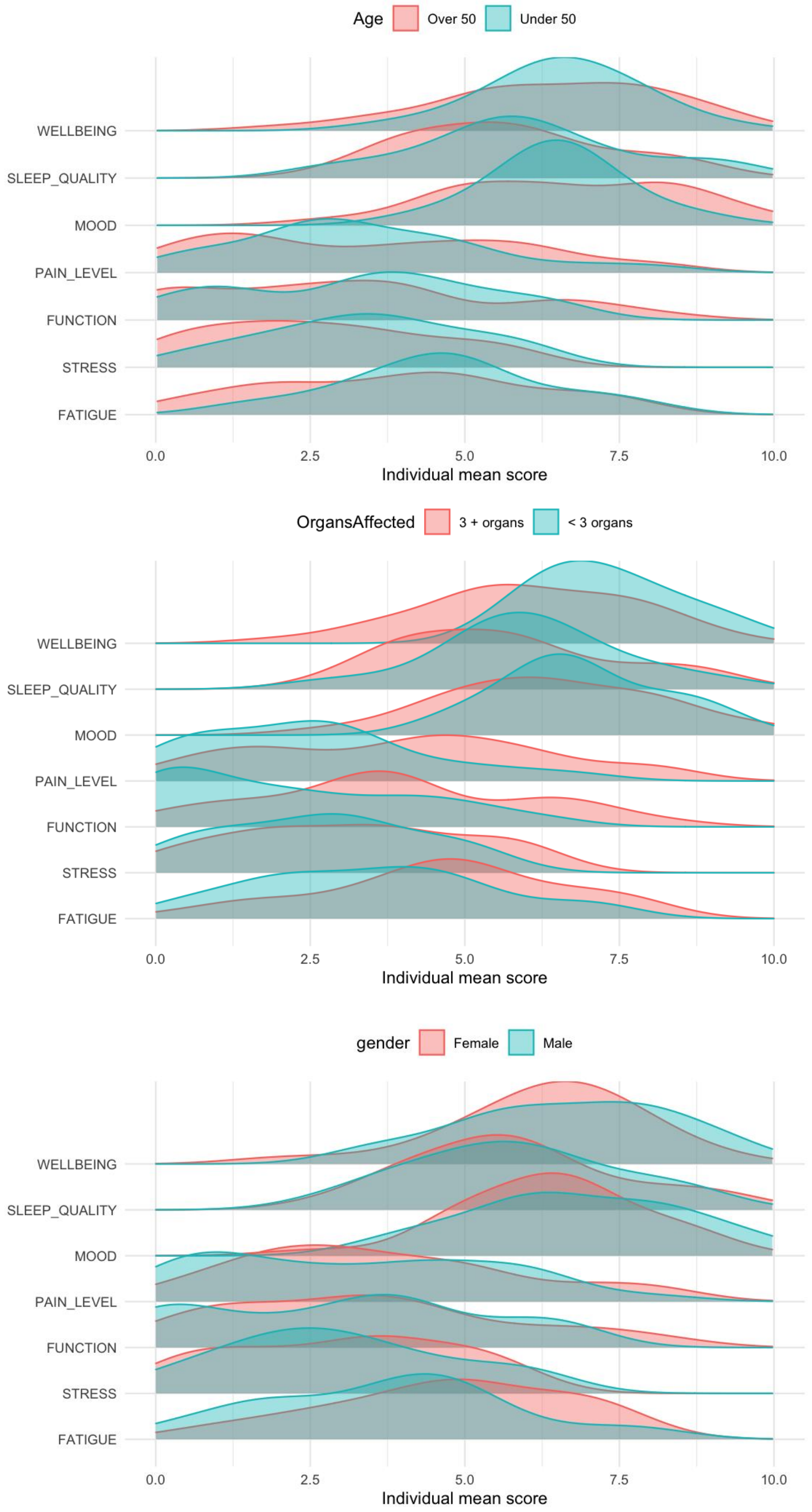

Supplementary Figure 3. *Wave plots illustrating the distribution of mean symptom ratings for individuals over the study period grouped by age, number of disease areas affected and Gender. For all symptoms other than mood, wellbeing and sleep quality a higher rating relates to a worse experience of that symptom. E.g. a pain score of 7 is more painful than score of 5.*

.

Supplementary Figure 4: *Dot plots illustrating the distribution of mean fatigue ratings over the study period coloured by the presence or absence of a co-morbidity affecting a given disease area.*

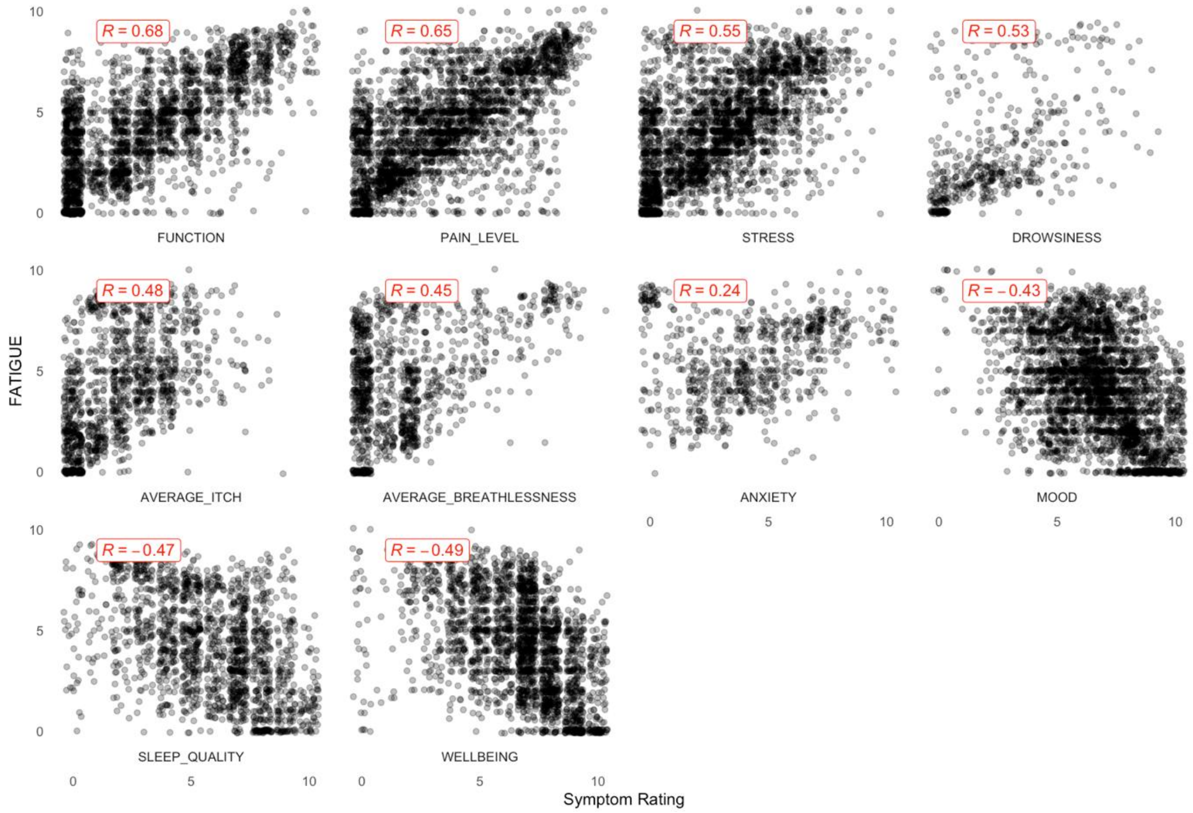

Supplementary Figure 5: *Scatterplot illustrating the correlation between fatigue and a given symptom for the whole cohort. Each dot represents a single individual on a given day. There are therefore multiple observations per participant.*

Supplementary Tables

Supplementary Table 1: List of survey questions

Question number	***Item name***	***Question stem***	***Time to trigger question All windows = 3h for daily, 1 day for weekly***	***Response type***	***Response options: Category options, or VAS anchors***
Baseline					
0.1	Employment	Are you currently employed?	On-boarding only	Binary	Y/N
0.2	Disease groups	Do you have a health condition affecting any of the following areas? (tick all that apply)	On-boarding only	Binary Y/N options for each variable, default to N<br>Tick all that apply	Bone, joint or muscle<br>Skin<br>Heart or lung<br>Stomach or bowel<br>Kidney<br>Mental health (anxiety)
Daily: Generic					
1.1	Sleep	How would you rate your sleep quality?	0800	VAS 0-10	0=Terrible<br>10=Excellent
1.2	Wellbeing	How well did you feel today?	1800	VAS 0-10	0=Very well<br>10=Very unwell
1.3	Pain	Overall level of pain today?	1800	VAS 0-10	0= No pain<br>10 = Worst possible pain
1.4	Mood	How is your mood?	0800<br>1200<br>1600<br>2000	VAS 0-10	0 = Very low<br>10-Very happy
1.5	Fatigue	How much fatigue do you feel?	0800<br>1200<br>1600<br>2000	VAS 0-10	0 = No fatigue<br>10=Extreme fatigue
1.6	Stress	How	1200	VAS 0-10	0 = Not at all stressed

		stressed are you?	1800		10=Extremely stressed
1.7	Function	How much difficulty have you had with daily tasks today?	1800	VAS 0-10	0=No difficulty<br>10=Extreme difficulty
Daily: Condition-specific					
1.10a (1.10a-e based on responses to 0.4)	Bone joint or muscle:<br>i) pain	What is your pain level now?	0800<br>1200<br>1600<br>2000	VAS 0-10	0= No pain<br>10 = Worst possible pain
	Bone joint or muscle:<br>ii) morning stiffness	How long were you stiff for this morning?	0800	Categorical	None<br>0-29 minutes<br>30-59 minutes<br>1-2 hours<br>2-4 hours<br>Over 4 hours
1.10b	Skin:<br>i) Average itch severity	How was your itch, on average in the past 24 hours?	2000	VAS 0-10	0 = no itch<br>10= worst imaginable itch
	ii) Worst itch severity	How was your worst itch in the past 24 hours?	2000	VAS 0-10	0 = no itch<br>10= worst imaginable itch
1.10c	Heart and lung<br>i) Average breathlessness	How was your breathing on average today?	2000	VAS 0-10	0=No breathlessness<br>10=Extreme breathlessness
	Heart and lung<br>ii) Worst breathlessness	How was your worst breathlessness in the past 24 hours?	2000	VAS 0-10	0=No breathlessness<br>10=Extreme breathlessness
1.10d	Stomach and bowels:<br>i) Appetite	How was your appetite today compared to normal?	2000	Categorical	Decreased a lot<br>Decreased a little<br>Unchanged<br>Increased a little<br>Increased a lot

	Stomach and bowels:<br>ii) Bowel habit	How were your bowel movements today?	2000	Categorical	1. Hard lumps, hard to pass/ Severe constipation;<br>2. Sausage shaped but lumpy/ Mild constipation;<br>3. Sausage shaped, surface cracks/ Normal<br>4. Like a sausage, smooth and soft/ Normal<br>5. Soft blobs, clear cut edges/ Lacking fibre<br>6. Fluffy pieces with ragged edges/ Mild diarrhoea<br>7. Watery, no solid pieces/ Severe diarrhoea<br><br>+ option 'No bowel movement'
1.10e	Kidney:<br>i) Drowsiness	Please select the number that best describes your drowsiness NOW (drowsiness = feeling sleepy)	0800<br>2000	VAS 0-10	0 = no drowsiness<br>10= worst possible drowsiness
	Kidney:<br>ii) Average itch severity	How was your itch, on average in the past 24 hours?	2000	VAS 0-10	0 = no itch<br>10= worst imaginable itch
	iii) Worst itch severity	How was your worst itch in the past 24 hours?	2000	VAS 0-10	0 = no itch<br>10= worst imaginable itch
1.10f	i) Mental health: Anxiety	How anxious were you today?	2000	VAS 0-10	0= Not at all anxious<br>10= Extremely anxious
